# The meta-memory ratio: a new cohort-independent way to measure cognitive awareness in asymptomatic individuals at risk for Alzheimer’s disease

**DOI:** 10.1186/s13195-020-00626-1

**Published:** 2020-05-14

**Authors:** Geoffroy Gagliardi, Marion Houot, Federica Cacciamani, Marie-Odile Habert, Bruno Dubois, Stéphane Epelbaum, for ADNI; for the INSIGHT-preAD study group, C. Audrain, A. Auffret, H. Bakardjian, F. Baldacci, B. Batrancourt, I. Benakki, H. Benali, H. Bertin, A. Bertrand, L. Boukadida, F. Cacciamani, V. Causse, E. Cavedo, S. Cherif Touil, P. A. Chiesa, O. Colliot, G. Dalla Barba, M. Depaulis, A. Dos Santos, B. Dubois, M. Dubois, S. Epelbaum, B. Fontaine, H. Francisque, G. Gagliardi, A. Genin, R. Genthon, P. Glasman, F. Gombert, M. O. Habert, H. Hampel, H. Hewa, M. Houot, N. Jungalee, A. Kas, M. Kilani, V. La Corte, F. Le Roy, S. Lehericy, C. Letondor, M. Levy, S. Lista, M. Lowrey, J. Ly, O. Makiese, I. Masetti, A. Mendes, C. Metzinger, A. Michon, F. Mochel, R. Nait Arab, F. Nyasse, C. Perrin, F. Poirier, C. Poisson, M. C. Potier, S. Ratovohery, M. Revillon, K. Rojkova, K. Santos-Andrade, R. Schindler, M. C. Servera, L. Seux, V. Simon, D. Skovronsky, M. Thiebaut, O. Uspenskaya, M. Vlaincu, F. Lamari

**Affiliations:** 1 Sorbonne Universités, UPMC Univ Paris 06, Inserm, CNRS, Institut du cerveau et de la moelle (ICM) - Hôpital Pitié-Salpêtrière, Paris, France; 2 AP-HP, Groupe Hospitalier Pitié-Salpêtrière, Département de Neurologie, Institut de la mémoire et de la maladie d’Alzheimer, Paris, France; 3 Centre of excellence of neurodegenerative disease (CoEN), ICM, CIC Neurosciences, APHP Department of Neurology, Hopital Pitié-Salpêtrière, University Paris 6, Paris, France; 4 Aramis project-team, Inria-APHP collaboration, F-75013 Paris, France; 5 Sorbonne Universités, CNRS, INSERM, Laboratoire d’Imagerie Biomédicale, Paris, France; 6 Centre pour l’Acquisition et le Traitement des Images, Paris, France; 7 Département de Médecine Nucléaire, AP-HP, Hôpital Pitié-Salpêtrière, Paris, France

**Keywords:** Awareness, Alzheimer’s disease, Preclinical

## Abstract

**Background:**

Lack of awareness of cognitive decline (ACD) has been described at the preclinical and prodromal stages of Alzheimer’s disease (AD). In this study, we introduced a meta-memory ratio (MMR) and explored how it is associated with neuroimaging AD biomarkers in asymptomatic individuals at risk for AD.

**Method:**

Four hundred forty-eight cognitively healthy participants from two cohorts of subjective memory complainers (INSIGHT-PreAD and ADNI) were included. Regression models were used to assess the impact of AD biomarkers on the MMR.

**Result:**

In both cohorts, there was a significant quadratic effect of cerebral amyloidosis on the MMR value. In particular, participants had a high ACD up to the amyloid positivity threshold, above which a decrease of ACD was eventually observed as the amyloid load increased.

**Conclusion:**

This nonlinear evolution of ACD in very early AD must be taken into account in clinical care and for trial enrollment as well.

## Introduction

It is now well known that the first brain changes due to Alzheimer’s disease (AD), the first lesions, and in particular amyloid aggregation, appear several decades before the clinical diagnosis of dementia [1–3]. In this preclinical phase, the accumulation of such lesions is accompanied by a subtle decline in cognitive domains, including executive functioning and memory [1, 4–8]. However, it has not yet been identified which measures, or combination of measures, would best predict the risk of decline to clinical AD for an asymptomatic individual [9]. Several studies have shown that the way individuals assess their own cognitive decline—the awareness of cognitive decline (ACD)—could be a promising measure.

On the one hand, the presence of cognitive complaints in otherwise cognitively normal (CN) elderly could increase the risk of decline to a later mild cognitive impairment (MCI) or AD dementia [10–14]. For instance, the prevalence of AD pathology has found to be higher in individuals with subjective cognitive decline (SCD), compared to those with no cognitive complaint [15–19]. However, the study of SCD as a risk factor for AD has led to inconsistent results, due to its non-specificity, as it may result from multiple etiologies [20, 21], and interact with the presence of other factors (e.g., depression, anxiety, personality, demographic factors and physical health concerns) [21].

On the other hand, anosognosia is frequently observed at the prodromal and dementia stages of AD [22]. There are at least three major ways to evaluate anosognosia in the literature [22–24]: (i) clinician rating of awareness, (ii) patient-informant discrepancy, and (iii) discrepancy between subjective and objective cognitive measures. In these methods, anosognosia would be defined as a gap between the subject’s perception of his/her own performance (overestimation) and a control measure (i.e., the clinician’s/caregiver’s rating of cognitive decline or the objective cognitive performance). Noteworthy, these methods still lack standardization [22]. Some studies have shown that impairment in ACD could occur before the clinical stage. Using the patient-informant discrepancy in asymptomatic at risks for AD subjects, we found that a higher level of AD biomarkers (i.e., amyloidosis and hypometabolism) in CN individuals was related to a low ACD [25]. Several other studies using the subjective-objective performance discrepancy approach showed low awareness of decline, both in asymptomatic individuals at risk for AD [26] and in individuals with MCI [24, 27–31]. Furthermore, Munro and colleagues [30] demonstrated that the presence of anosognosia could predict future conversion from MCI to clinical AD. There exists consistent evidence that anosognosia would be associated to mainly frontal but also temporoparietal dysfunctions, notably in the regions involved in self-referenced treatment and memory [26, 28], as well as to a disconnection between these same areas [27]. Similarly, anosognosia could be linked to the amount of cerebral amyloidosis in individuals with MCI [31, 32].

Recently, Vannini and colleagues [32] proposed a chronological model of the evolution of ACD in preclinical AD. Awareness disorders would begin at the preclinical stage with a hypernosognosia (otherwise called SCD), subsequently turning into a low ACD alongside the onset of subtle cognitive changes, and finally into anosognosia in the presence of increasing AD brain lesions.

Thus, although anosognosia is a well-known symptom of late-stage AD, it is currently debated whether SCD or a decreased ACD is better associated with AD brain lesions and the subsequent cognitive decline at the pre-dementia stages.

Studies on preclinical AD are currently suffering from a lack of standardized evaluation protocols [33]. The diversity of methodologies to compute and analyze ACD at these early stages, together with the small samples, may participate to obfuscate the comprehension of the chronology of awareness disorders.

In our study, we tried to address this issue by designing a new methodology to measure the ACD that can be applied to any cohort using different cognitive tests, in order to facilitate inter-cohort comparisons. We introduced the meta-memory ratio (MMR) and applied it in two different cohorts of asymptomatic individuals at risk of AD (i.e., ADNI and INSIGHT-PreAD). According to the model proposed by Vannini and colleagues [32], we expected that the degree of awareness will increase with the level of AD lesion load, and then gradually decrease.

## Materials and methods

### Participants

We analyzed data from two cohorts, INSIGHT-PreAD and ADNI. The INSIGHT-PreAD cohort [34] is a French mono-centric cohort of cognitively normal elderly memory complainers, longitudinally followed at the Pitié-Salpêtrière Hospital (Paris). At baseline, the sample included 318 participants aged between 70 and 85 years old, with Mini-Mental State Examination (MMSE) [35] ≥ 27/30, total recall of the Free and Cued Selective Reminding Test (FCSRT) ≥ 41/48, and Clinical Dementia Rating (CDR) = 0.

For the present study, some participants were excluded from the original sample (i.e., one participant due to missing metabolic imaging, one due to missing memory scores, 24 due to missing questionnaires). In addition, two outliers (i.e., one on a memory score, the other on brain metabolism) were also removed from the sample. Our final sample included 290 participants from the INSIGHT-PreAD cohort.

The Alzheimer’s Disease Neuroimaging Initiative (ADNI, http://adni.loni.usc.edu) is a multicentric longitudinal study. For this study, we aimed at including only ADNI participants with SCD (i.e., tagged as Significant Memory Concern), in order to be fully comparable to INSIGHT-PreAD participants. These are subjects with normal cognition (MMSE ≥ 24/30; Logical Memory Delayed Recall in standards, CDR = 0) and memory concerns not supported by the informant (Cognitive Change Index, sum of the first 12 items > 12/16) [36]. We identified 277 participants meeting these criteria. We excluded the participants with missing data, and a total of 158 participants from ADNI cohort were retained in the final sample.

For each cohort, we used only baseline data. Our final sample therefore consisted of 448 CN participants with SCD.

### Development of the MMR

#### Objective memory assessment

In this study, we have chosen to focus on memory. Indeed, recent research tends to show that the subtle decline in memory occurring in preclinical AD would be among the earliest to evidence of a transition to a subsequent prodromal AD [1, 5–8].

Therefore, we selected three episodic memory scores: the Free and Cued Selective Reminding Test (FCSRT) [37], the Delayed Matched to Sample test 48 items (DMS48) [38], and the Rey-Osterrieth Complex Figure (ROCF) [39]. For the FCSRT, we selected both immediate and delayed total free recalls (FR) and total recalls (i.e., FR + cued recalls; TR), the number of intrusions and perseverations. For both visual tests (i.e., DMS48 and ROCF), we used immediate and delayed memory measures. Details of the neuropsychological examination proposed in the INSIGHT-PreAD cohort were previously described [34].

For the ADNI cohort, we selected three memory tests, namely the Rey Auditory Verbal Learning Test (RAVLT, “immediate,” “forgetting,” and “learning” scores) [40], the Logical Memory II (LMII) test from the Wechsler Memory Scale [41], and the Q4 (memory) score from the Alzheimer Disease Assessment Scale (ADAS-Cog) [42].

#### Subjective cognitive assessment

For INSIGHT-PreAD cohort, we used the cognitive subscale of the Healthy Aging Brain Care – Monitor questionnaire [43]. This questionnaire asks subjects to rate the frequency of occurrence of certain cognitive disturbances during the last two weeks (i.e., from 0 “not at all (0–1 day)” to 3 “almost daily (12–14 days)”). The HABC-M cognitive scale consists of 6 items, the majority of which are related to the memory domain. The total score ranges from 0 to 18.

For the ADNI cohort, we used the memory subscale of the Everyday Cognition questionnaire score [44]. These questions ask the participant to compare his/her current memory ability in everyday tasks to that of 10 years ago. The estimate is based on a 4-point scale from 1 (“Better or no change”) to 4 (“Consistently much Worse”). A “Do not know” answer is also possible. The total score then ranges from 8 to 32. Higher scores indicate that the participant perceives a more marked cognitive decline.

#### The meta-memory ratio

**Fig. 1:**
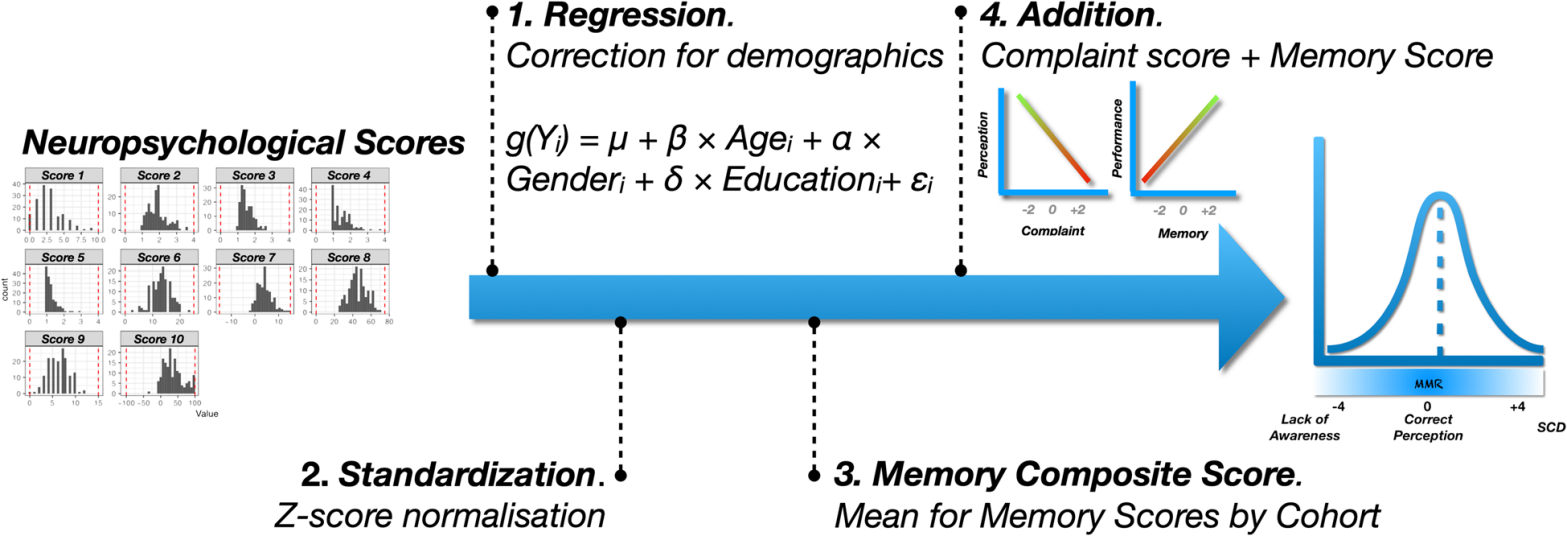
MMR’s construction

We based our measure of ACD on the model of the “anosognosia index” initially proposed by Dalla Barba and collaborators [45]. This procedure consists in measuring a gap between the subjective complaint and an objective performance. To compute the score, the same procedure was implemented independently in each cohort (Fig. 1).

First, since the two samples had different demographic distributions and these variables can be associated with the scores of interest, we started by removing their impact on the scores. Each score of interest (i.e., memory performances and complaint questionnaires) was integrated into a generalized linear model (GLM), as a dependent variable. Demographic variables (i.e., age, gender and socio-cultural level) were included as covariates, to correct for their potential effects. For each measure, the type of model used was selected according to the distribution of each score (i.e., linear regressions for ROCF, ECog, immediate RAVLT, and ADAS-Q4; logistics for FCSRT intrusions and perseverations; and binomial for the other measures mentioned). Subsequently, we extracted the model residuals to obtain objective and subjective measures of decline net of these effects.

Secondly, we have centered and reduced all the residuals in order to make them comparable (i.e., *z*-score transformation).

Thirdly, we computed a composite score by averaging all memory scores collected for each subject. In this way, we had two values. The first one represented an objective measure of memory function. The higher this score, the better the memory performance at testing. The second one represented a subjective measure of memory. The higher this score, the higher the memory complaint.

The choice of relying on a composite score rather than choosing a single memory score addressed two needs. To begin with, it allowed us not to select a certain score a priori. In addition, the use of a composite score also allowed to gather variables that are thought to measure the same cognitive construct [46]. We would have used this procedure also for complaint measures, if the cohorts had included more than one questionnaire.

Finally, we added these two scores. By construction, an MMR close to 0 corresponds to a good match between subjective rating and objective performance (i.e., accurate ACD). The higher the MMR is, the more it corresponds to an SCD (i.e., important complaint with correct performance). On the contrary, the lower it is, the more the ACD is low (i.e., lower complaint associated with a poorer performance; Fig. 1).

### Brain imaging acquisition and processing

#### Amyloid PET imaging

In the INSIGHT-PreAD cohort, participants underwent PET with a florbetapir tracer [18F-Florbetapir, Amyvid™, Avid Radiopharmaceuticals]. A standardized uptake value ratio (SUVr) was calculated with the CATI pipeline (Centre d’Acquisition et de Traitement d’Images, https://cati-neuroimaging.com), with a focus on selected target regions (i.e., bilateral precuneus, anterior and posterior cingulum, temporal cortex and orbitofrontal). Details of the imaging procedure and threshold calculations have previously been presented [34, 47]. For ADNI participants, we selected those with SUVr values calculated using the same radiotracer (i.e., florbetapir). The details of both imaging procedures are presented in supplementary materials.

To make the two cohorts neuroimaging features comparable, we normalized the SUVr using the respective amyloid positivity threshold of each cohort. To do so, we divided the SUVr of each participant by the positivity threshold, i.e., 0.79 for the INSIGHT-PreAD cohort and 1.11 for ADNI. Thus, any normalized SUVr above 1 could be considered significantly pathological (i.e., amyloid-positive patients).

#### FDG-PET imaging

For each of our cohorts, we calculated a mean metabolism index using fluorodeoxyglucose positron emission tomography (FDG-PET) by averaging the regions of interest (ROIs) of AD, namely the posterior cingulate cortex, inferior parietal lobule, precuneus, and inferior temporal gyrus [48]. Then, since the FDG-PET did not have an established cutoff, we normalized this meta-ROI using a centered-reduced method (i.e., intra-cohort *z*-score transformation). As for amyloid PET imaging, details are available in supplementary materials.

### Statistical analysis

The different scores of interest (MMR, complaint and memory) and demographic variables were compared between the two cohorts using Welch’s *t* tests for the numerical variables and a *χ*^2^-test for the gender variable.

MMR scores were normally distributed. In order to evaluate the influence of AD biomarkers on awareness, we computed a linear regression model with the MMR as dependent variable. To account for the specific effect of each biomarker, amyloidosis (AV45-PET) and metabolism (FDG-PET) were both included in the model. We also included interactions between the “cohort” effect and biomarkers to determine whether the effect of biomarkers varied across cohorts. Finally, we adjusted the results including demographic (i.e., age, gender and education) and the cohort variables as covariates.

Looking at the scatterplot between MMR and AV45-SUVr, we identified a non-linear effect of amyloid on the MMR. Therefore, we added a quadratic effect of amyloid to the models. The main effects and interactions (both with linear and quadratic effect) were tested via the likelihood ratio test type II. Normality of residuals and heteroskedasticity were checked visually. Cook’s distances and hat values were computed to investigate potential influencers and outliers. We also performed these computations with an additional group of cognitively normal (CN, that is normal cognition without cognitive complaint) from ADNI without anosognosia (data not shown). Finally, the same analysis was performed with an MMR calculated from a single rather than a composite memory score. These results can be found in supplementary material (Additional File 3).

Statistical analyses were performed using R 3.5.2 (https://www.R-project.org/). An R package was developed for the calculation of MMR in various cohorts (https://github.com/GagGeo/MMAD).

## Results

### Inter-cohort comparisons

ADNI participants were younger (72.0 ± 5.8 vs 76.0 ± 3.5, *p* < 0.001) and had higher education (99.4% vs 67.6%, *p* < 0.001) compared to INSIGHT-PreAD participants (Table 1). More details can be found in Supplementary Materials.

**Table 1:** Differences between cohorts

	ADNI (***N*** = 158)	INSIGHT-PreAD (***N*** = 290)	T/ChiSq	***P***val
**Age**	71.97 ± 5.79	76.02 ± 3.5	-8,03	<0.001*
**Gender (F)**	95 (60.1%)	183 (63.1%)	0,27	0.604
**Education (H)**	157 (99.4%)	196 (67.6%)	59,94	<0.001*
**AV45**	1.01 ± 0.16 [0.77;1.56]	0.86 ± 0.17 [0.65;1.54]	9,42	<0.001*
**FDG**	0 ± 1 [-2.69;2.94]	0 ± 1 [-2.49;3.78]	0	1.000
**Memory**	0 ± 0.38 [-0.93;1.41]	0 ± 0.54 [-1.97;1.23]	0,04	0.970
**Complain**	0 ± 1 [-1.86;3.21]	0 ± 1 [-1.26;3.41]	0	1.000
**Informant**	0 ± 1 [-1.31;4.41]	0 ± 1 [-0.97;4.59]	0	1.000
**MMR**	0 ± 1.07 [-2.44;3.61]	0 ± 1.08 [-2.15;3.11]	0,02	0.988

Mean ± standard-deviation [minimum ; maximum]. *MMR* meta memory ratio

**Fig. 2:**
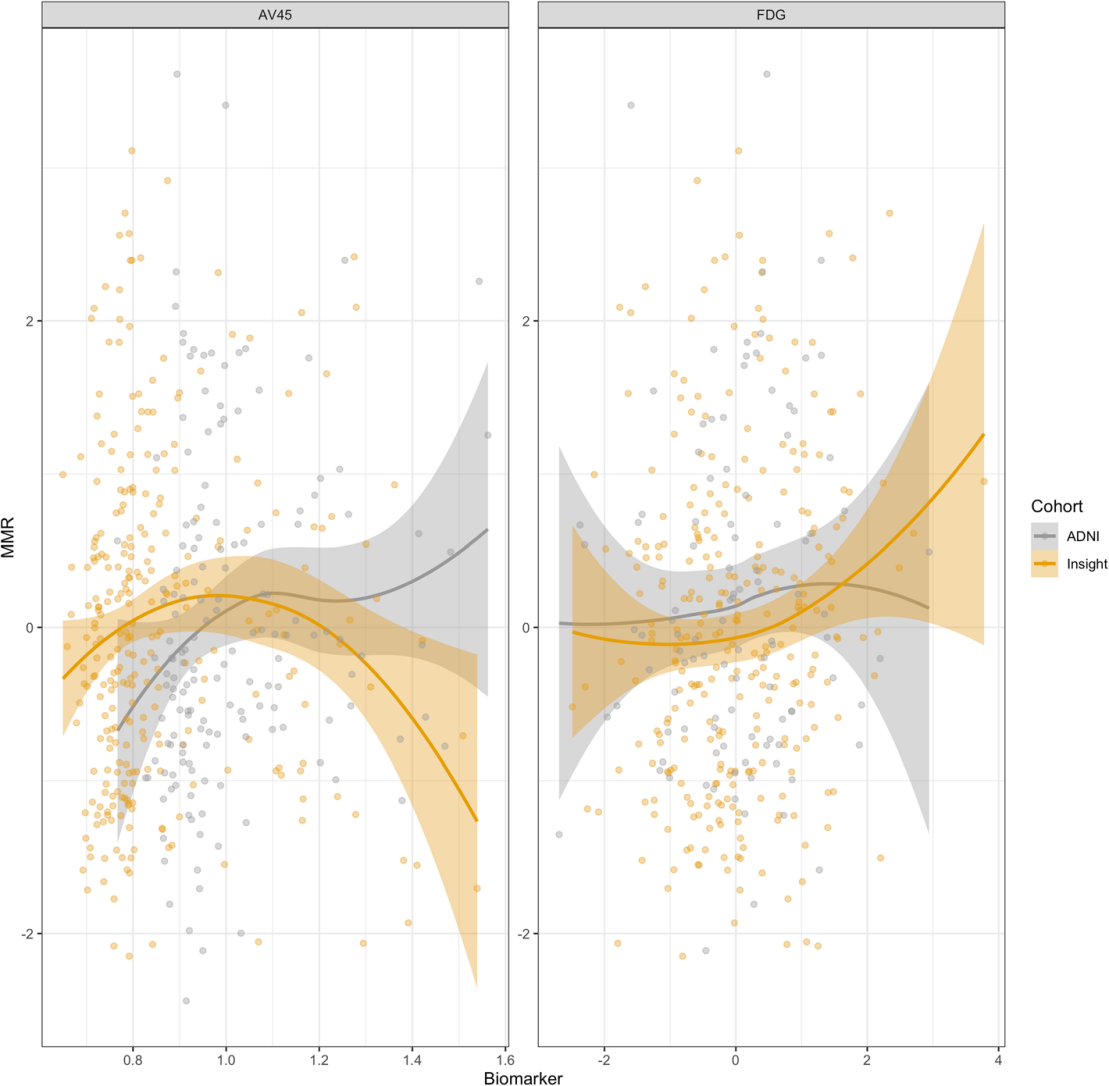
Comparison of the relative effect of biomarkers by cohort. MMR, meta-memory ratio; FDG mean metabolism, computed using FDG-AD ROIs

ADNI subjects also had a significantly higher amyloid load than INSIGHT-PreAD subjects (1.0 ± 0.2 vs 0.9 ± 0.2, *p* < 0.001). Overall, these imaging variables appeared to be normally distributed across the two cohorts (Fig. 2).

### MMR models

**Fig. 3:**
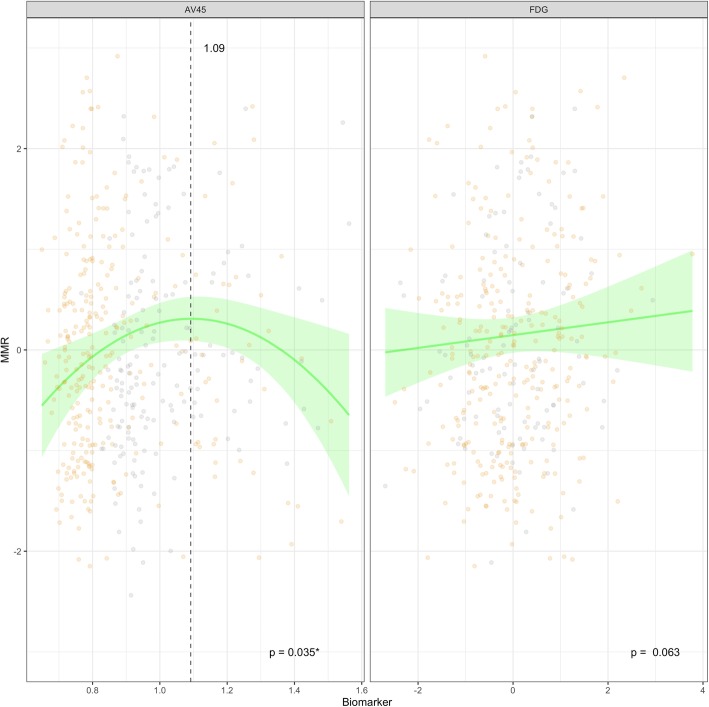
Effect of biomarkers on MMR score

The model (Fig. 3) showed a slight association between metabolism and awareness measures. In particular, the MMR decreased with decreasing brain metabolism. This trend, however, was not statistically significant (*p* = 0.063). Regarding the AV45 PET value, we found a significant combined (linear and squared; *p* = 0.035) effect on the MMR score. In the curve of this quadratic effect, an inflection point is observed at a normalized SUVr value of 1.09.

We did not find any significant differences of the cohort variable nor for the interactions (all *p* > 0.05; Fig. 2), indicating that the effect of the biomarkers was not statistically different on MMR in the two cohorts (Table 2).

**Table 2:** Results of linear models on MMR

Measures	Coefficients ± SE	ESs	***P***value
**Intercept**	-4.67 ± 2.3	<0.001	
**Age**	-0.01 ± 0.01	<0.001	0.267
**Gender (M)**	0.03 ± 0.12	<0.001	0.714
**Education (Lower)**	0.13 ± 0.14	<0.001	0.334
**Cohort (ADNI)**	1.51 ± 0.92	<0.001	0.385
**FDG**	0.96 ± 1.38	<0.001	0.063
**AV45**		-0.007	0.035*
**Linear**	10.31 ± 3.69		
**Squared**	-4.37 ± 1.6		
**AV45:FDG**	0.87 ± 1.26	<0.001	0.787
**AV45:Cohort (ADNI)**	-1.52 ± 0.91	<0.001	0.578
**FDG:Cohort (ADNI)**	0.01 ± 0.15	<0.001	0.450

Coefficients and standard error (S.E.) were extracted from complete LMs with all interactions. For each categorical effect, the reference category is given in bracket. *S.E.* Standard Error, *ESs* Effect Sizes (Cohen's F2)

When a CN group without cognitive complain was added, the results were also not showing any variable effect between the different groups. However, this significant effect (AV45) and trend (FDG) was no longer present, as the addition of the no-complaint group masked the effects observed in the previous samples (all *p* > 0.05; data not shown). Finally, the analysis carried out with an MMR constructed on the basis of a single rather than a composite memory score did not show significant results (all *p* > 0.05; Additional File 3, Table 3).

## Discussion

In this study, we had two distinct objectives. On the one hand, we aimed to determine the chronology of variations in awareness in the early stages of AD. On the other hand, we aimed to develop a new method to assess the ACD that can be applied in several SCD cohorts, in order to obtain more generalizable results. We have developed the meta-memory ratio (MMR), which provides a continuous measurement of the awareness of one’s own memory performance. We implemented it in two samples, the INSIGHT-PreAD cohort and SMC subjects from ADNI, in order to assess its trans-cohort applicability. To assess its applicability on a trans-cohort perspective, we implemented the MMR in two separate samples, the INSIGHT-PreAD and ADNI cohorts. MMR has the advantage of being easy to compute (with an R package available) and is potentially applicable in any cohort that has at least a cognitive score and a self-rating measure of cognitive functioning.

Regarding amyloidosis, it was normalized using cohort positivity thresholds. This method is less accurate than the Centiloid scale [49]. However, Habert and colleagues [47] showed a strong correlation between the Avid (used in ADNI) and CATI (used in INSIGHT-PreAD) methods (*r* = 0.9). We thus considered our SUVr normalization as an acceptable approximation, with improved processing time and simplicity.

The regression between MMR and AD biomarkers (i.e., amyloidosis and brain metabolism) showed a significant quadratic effect of amyloidosis: the MMR scores increased, indicating a complaint without objective decline, with increasing amyloid load, up to a certain threshold, above which the increase in amyloid load was associated with a lower MMR score, indicating a decline in the ACD. This is consistent with previous studies indicating that both SCD and low ACD could be associated with a greater risk of AD pathology [10–14, 25]. These findings may appear contradictory. However, they can be understood as two successive chronological phases. Indeed, our results are in line with the aforementioned study by Vannini and colleagues [32] proposing that, at the preclinical stage, a hyper-vigilance towards otherwise undetected cognitive difficulties (i.e., hypernosognosia, SCD) would precede subsequent low ACD. Individuals with SCD would be at an early stage of the disease, before the decrease in ACD. Interestingly, with the inflection point at 1.09, it seems that the complaint progresses into a low and decreasing ACD when the participants become amyloid positive. Previous studies had already shown a link between amyloidosis and anosognosia in MCI populations [31, 32]. The present study demonstrated this relationship in CN individuals.

These results have strong implications in both research and clinical practice. Indeed, some consider that the appearance and aggravation of a complaint could be used as a marker of risk of having AD lesions [10–14]. However, our study showed that the accumulation of amyloidosis initially leads to increasing complaints, eventually turning into poor awareness. Thus, the presence of persistent cognitive complaints should not be considered as indicative of AD, but due to other etiologies [20, 21]. Even in the presence of amyloid accumulation, cognitive complaints should be considered as a minor risk for AD. Indeed, some studies showed an increase in amyloid burden with advancing age [50], regardless of whether or not there is a subsequent conversion to AD. Demonstrating that the decrease in awareness takes place beyond the amyloid-positivity threshold, it seems that low awareness, rather than complaints, should be taken as marker of AD. Taken together, our results seem to validate the chronological models mentioned above, which assumes an increase and then a decrease in the ACD during the evolution towards the diagnosis of AD [32, 51].

In the present study, we also identified a slight (not significant) effect of metabolism on ACD (i.e., higher metabolism resulting in a higher MMR score). There are several possible reasons. To begin with, since amyloid is the first biomarker to accumulate [52], hypometabolism only occurs later. Demonstrating a relationship between amyloidosis but not neurodegeneration (represented here by hypometabolism), these data could mean that impairments in consciousness of the disorders may be sensitive earlier than variations in brain metabolism.

In addition, we analyzed mean metabolic indices in AD ROIs, selected from previous research for their sensitivity and specificity in clinical AD [48]. However, deficits in ACD could not be associated with these same brain regions. Awareness has often been associated with cortical midline structures [27, 28, 32], as well as right prefrontal regions [53, 54]. For instance, a recent study conducted as part of the INSIGHT-PreAD study found that low awareness was related to lower brain metabolism in a fronto-temporo-parietal network [25]. This network does not overlap with the ROIs considered in this study. Traditionally, AD symptoms have been attributed to tauopathy [55]. However, at this preclinical stage, tauopathy is mainly localized in the mesial temporal regions [56], and it is probably subtle given the absence of significant memory deficits. Amyloidosis, on the other hand, begins to accumulate upstream [3, 52, 57] notably in prefrontal regions [58] which seems to be related to awareness [25, 53, 54]. This may explain the stronger relationship between MMR and amyloid load than with metabolism. Nevertheless, further longitudinal studies are needed to conclude on the neural correlates of the decline in ACD.

In this study, we compared objective performance with the subjective perception of participants. As explained above, other methods exist to evaluate ACD [22, 23]. In a previous study with asymptomatic individuals, we had shown that a discrepancy between the subject’s and informant’s perceptions resulted in higher levels of biomarker accumulation [25]. Few studies have attempted to compare awareness measures [24], and the question arises as to what would be the best tool to measure a weakening of the ACD. It would be interesting to directly compare the MMR methodology to existing assessment methods.

In this work, we selected participants with subjective cognitive complaint and normalized their scores (subjective and objective) on the basis of the sample they belonged to. As a result, some participants could obtain a low relative complaint score. Similarly, we interpret lower scores on the MMR as signs of low ACD. It could be suggested that low ACD is inconsistent with the presence of SCD. However, ACD is defined in relation to the reference sample. Moreover, using the participant-informant discrepancy, Hanseeuw et al. [59] show that although the subject’s complaint is initially higher than the informant’s, the gap between the two narrows to a reversal of this ratio about 1.6 years before the MCI diagnosis. Thus, there would be a period in which, although ACD has begun to decline, the participant can still be defined as SCD. It should also be noted that in our study, there was no significant effect of AD biomarkers on isolated complaints from the participant or informant (see Additional File 3, Table 1).

Additionally, the model performed with an MMR calculated on the basis of a single memory score does not highlight this significant effect of amyloidosis (see Additional File 3, Table 3). It is possible that a composite score would better reflect the whole memory process, whereas a single score could be “parasitized” by the involvement of other cognitive components (e.g., executive) that are more fragile and subject to various external or internal factors.

There are some limitations to our study. First, despite our efforts to make the measures comparable with each other, the scores used to compute the MMR are not exactly the same in the two cohorts, and they might involve slightly different cognitive processes. Moreover, the cohorts varied on two of the three demographic variables considered, with INSIGHT-PreAD participants being older and proportionally less educated. Among the participants in the ADNI cohort, only one individual had a level of education of less than 12 years. This over-representation of graduates does not seem to be in line with the general population proportions, which raises the question of the generalization of the results. Indeed, the research on the concept of cognitive reserve has shown a significant effect of schooling on the clinical expression of brain damage [60–62]. Similarly, a higher proportion of positive amyloid participants (i.e., normalized AV45-SUVr ≥ 1) is found in ADNI than in INSIGHT-PreAD. Finally, the construction of the MMR involved a pre-processing of the data whereby participants were ranked according to their relative performance in their sample. In doing so, it was assumed that individuals with low levels of functioning might show a decline from normal prior function. However, despite the normalization of scores, it is possible that these are simply life-long low performers. This limitation could be controlled by a longitudinal study of the MMR and its evolution.

## Conclusion

The MMR has demonstrated its trans-cohort applicability. Using this methodology, we observed a quadratic relationship between ACD and cerebral amyloidosis. The more cerebral amyloidosis our participants had, the more they tended to present an SCD up to a certain threshold beyond which, on the contrary, this tendency reverses to lead individuals to low ACD. Future studies should include the MMR in longitudinal analyses to focus on the ACD evolution and to validate this chronological model. Similarly, further studies are needed to determine the sensitivity and specificity for AD of decreased ACD versus SCD. This question can be examined both through the use of research cohorts (e.g., ADNI, INSIGHT-PreAD), clinical cohorts (e.g., Subjective Cognitive Impairment Cohort – SCIENCe), or in general population studies (e.g., Wisconsin Registry for Alzheimer’s Prevention – WRAP–, Mayo Clinic Study of Aging – MCSA). Furthermore, currently, most ACD studies use the opposition between the participant’s complaint and the informant’s complaint. Few studies have attempted to compare different methods of assessing awareness in this type of early population. This question may be the subject of further work to determine which method would first demonstrate an inflection of ACD in longitudinal follow-up (in preparation).

Overall, our results promoted the value of assessing ACD in elderly CN individuals as a measure of the risk of conversion to later clinical AD. Therefore, the inclusion of a measure of ACD would be valuable in cohorts targeting preclinical AD as an enrichment variable.

## Supplementary information

**Additional file 1.** Additional methods. Description of the procedures for acquisition and processing of imaging measurements.

**Additional file 2.** Data distribution of the samples. Population distribution regarding measures of interest (i.e. cognition, imaging and MMR).

**Additional file 3.** Additional results for the presented model, and presentation of results with variations in the MMR construction.

## Data Availability

Requests for access to the original data should be addressed to the study sponsors. The ADNI data are available at http://adni.loni.usc.edu. The INSIGHT-PreAD study data are available at https://www.gaaindata.org/partner/INSIGHT-preAD. Access rights are subject to the submission of a project. Please contact B. Dubois at bruno.dubois@aphp.fr.

## Abbreviations

ACD: Awareness of cognitive decline
AD: Alzheimer’s disease
ADAS: Alzheimer Disease Assessment Scale
ADNI: Alzheimer’s Disease Neuroimaging Initiative
CATI: Centre d’Acquisition et de Traitement d’Images
CN: Cognitively normal
DMS48: Delayed Matched to Sample test 48 items
ECog: Everyday Cognition questionnaire score
FCSRT: Free and Cued Selective Reminding Test
FDG: Fludeoxyglucose
FR: Free recall
GLM: Generalized linear model
HABCM: Healthy Aging Brain Care – Monitor questionnaire
INSIGHT-PreAD: INveStIGation of AlzHeimer’s PredicTors in subjective memory complainers – Pre Alzheimer’s disease
LMII: Logical Memory II
MCI: Mild cognitive impairment
MCSA: Mayo Clinic Study of Aging
MMR: Meta-memory ratio
PET: Positron emission tomography
RAVLT: Rey Auditory Verbal Learning Test
ROCF: Rey-Osterrieth Complex Figure
ROI: Regions of interest
SCD: Subjective cognitive decline
SCIENCe: Subjective Cognitive Impairment Cohort
SUVr: Standardized uptake value ratio
TR: Total recall
WRAP: Wisconsin Registry for Alzheimer’s Prevention
