# Supplementary material for: The meta-memory ratio: a new cohort-independent way to measure cognitive awareness in asymptomatic individuals at risk for Alzheimer’s disease
Source: Alzheimers Res Ther. 2020 May 14;12:57. doi: 10.1186/s13195-020-00626-1 (PMC7222501; doi:10.1186/s13195-020-00626-1)
Supplement: Supplementary file 1 — Additional file 1. Additional methods. Description of the procedures for acquisition and processing of imaging measurements. [file 13195_2020_626_MOESM1_ESM.pdf]

Additional Methods

Amyloid PET Imaging

Regarding the INSIGHT-PreAD cohort, as it described in Habert and colleagues' article [41], all amyloid PET were acquired in a single session on a Philips Gemini GXL CT-PET scanner. The scan was performed approximately 50 (± 5) min after the injection of ^{18}F-Florbetapir (AVID radiopharmaceuticals ; ~ 370 MBq, range 333–407 MBq). The acquisition consisted of 3 × 5-min frames, in a 128 × 128 acquisition matrix (voxel size of 2 × 2 × 2 mm^3). The LOR-RAMLA algorithm was used for reconstruction (10 iterations). Reduction of noise modulated the relaxation parameter lambda, which was set at 0.7. All corrections (attenuation, scatter and random coincidence) were integrated in the reconstruction.

For ADNI participants, we selected those with SUVr values calculated using the same radiotracer (i.e. Florbetapir). The cortical ROI used was calculated using four grey matter ROI (i.e. frontal, anterior/posterior cingulate, lateral parietal and lateral temporal) and five reference regions (cerebellar grey matter, whole cerebellum, brainstem/pons, eroded subcortical white matter, and a composite reference region). We then used the average AV45 SUVR of frontal, anterior cingulate, precuneus, and parietal cortex relative to the whole cerebellum.

FDG-PET Imaging

For the INSIGHT-PreAD participants, the same pipeline as for amyloid was applied on ^{18}F-FDG PET scans. We calculated cortical metabolic values within four bilateral regions, i.e. posterior cingulate cortex, inferior parietal lobule, precuneus, and inferior temporal gyrus. Those regions were selected as specifically early sensitive AD. Regarding the reference region, we used the pons.

For ADNI, we used the value calculated from five MetaROI volumes selected to be sensitive to early AD changes, i.e. the angular gyrus (left and right), the posterior cingulate (bilateral), and the inferior temporal gyrus (left and right).
