# Supplementary material for: The meta-memory ratio: a new cohort-independent way to measure cognitive awareness in asymptomatic individuals at risk for Alzheimer’s disease
Source: Alzheimers Res Ther. 2020 May 14;12:57. doi: 10.1186/s13195-020-00626-1 (PMC7222501; doi:10.1186/s13195-020-00626-1)

Additional Figures

1. Histograms: Biomarker Raw Distributions

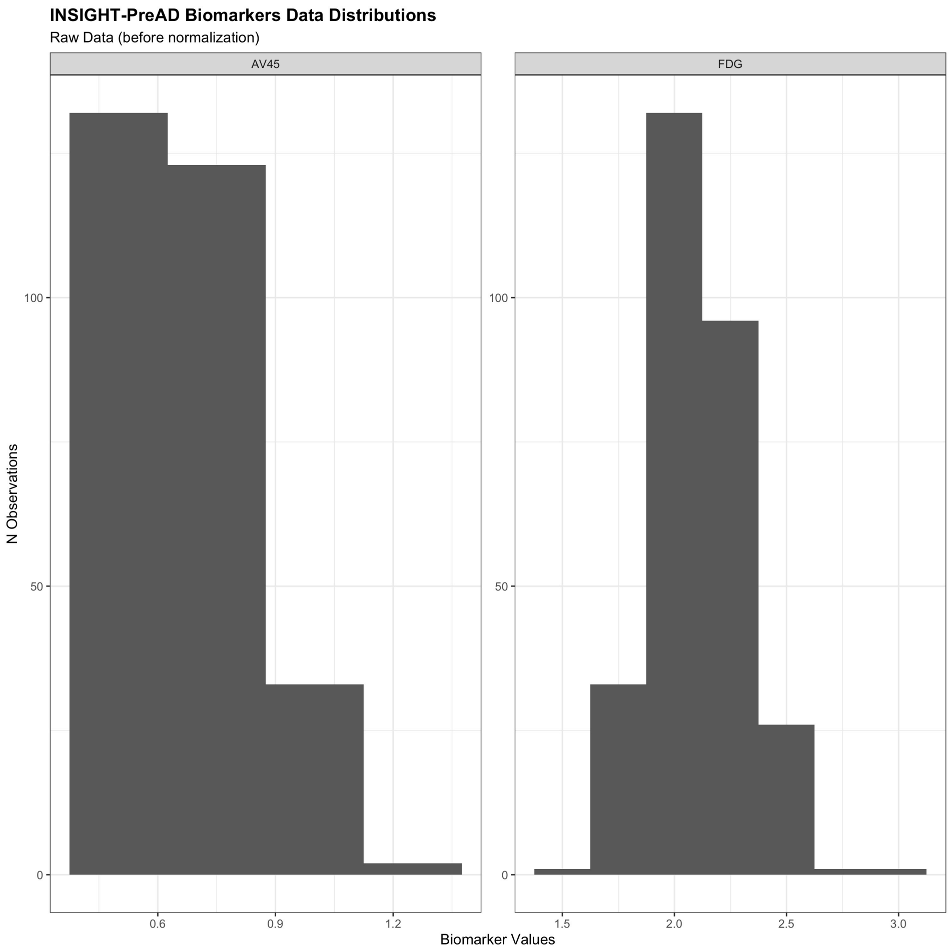

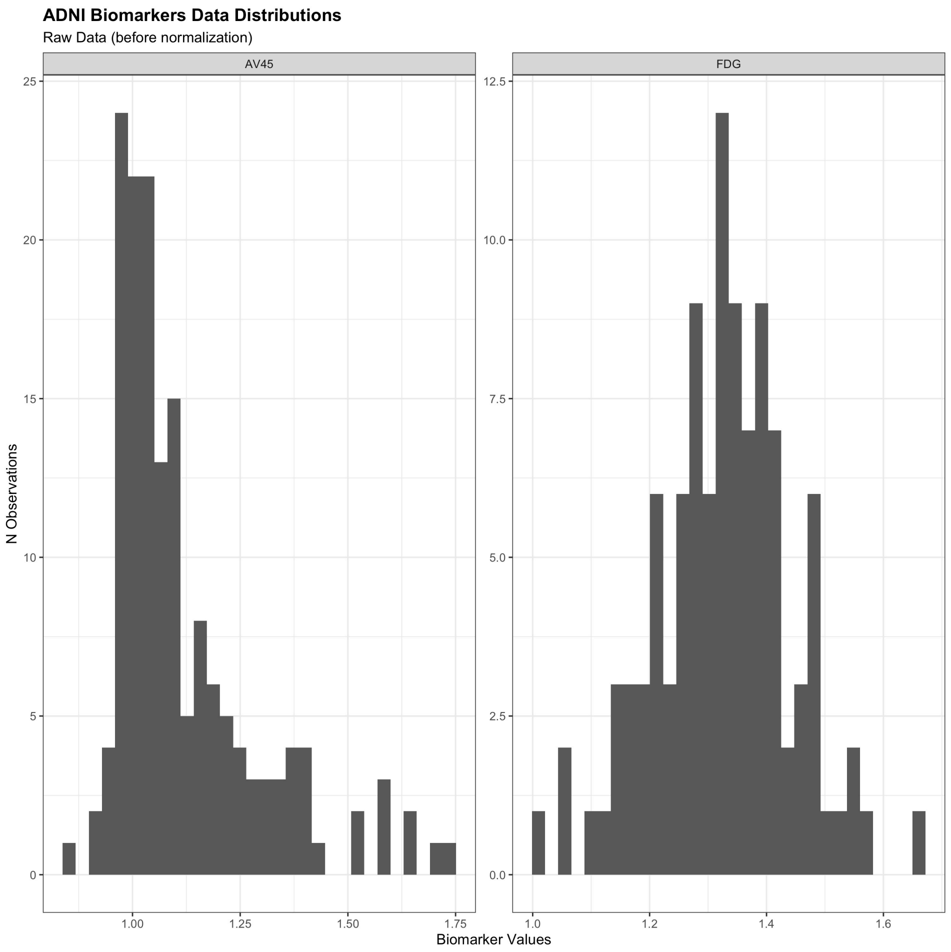

2. ADNI, Neuropsychological Variables, Raw Distributions

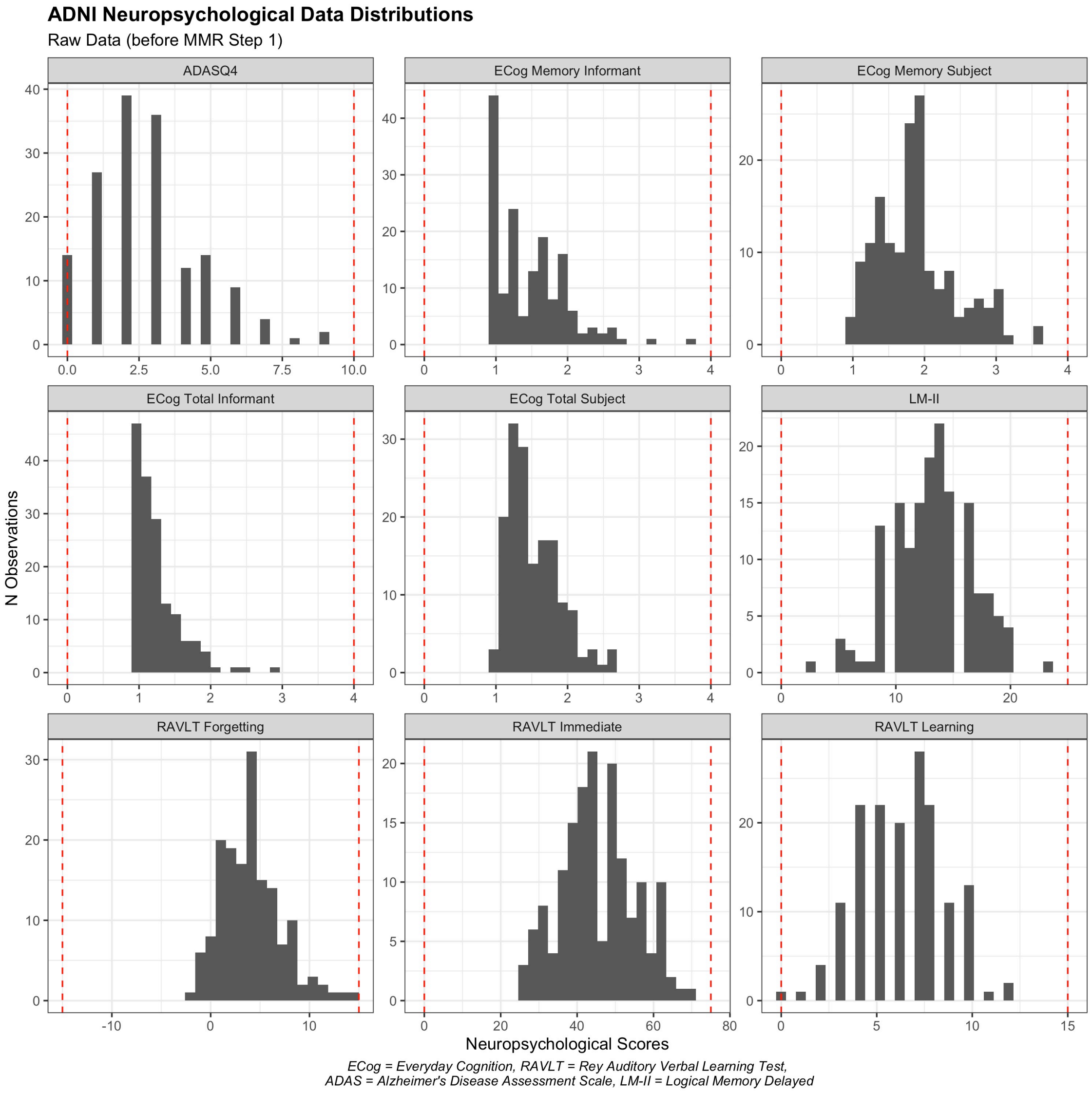

3. ADNI, Neuropsychological Variables, Transformed Distributions

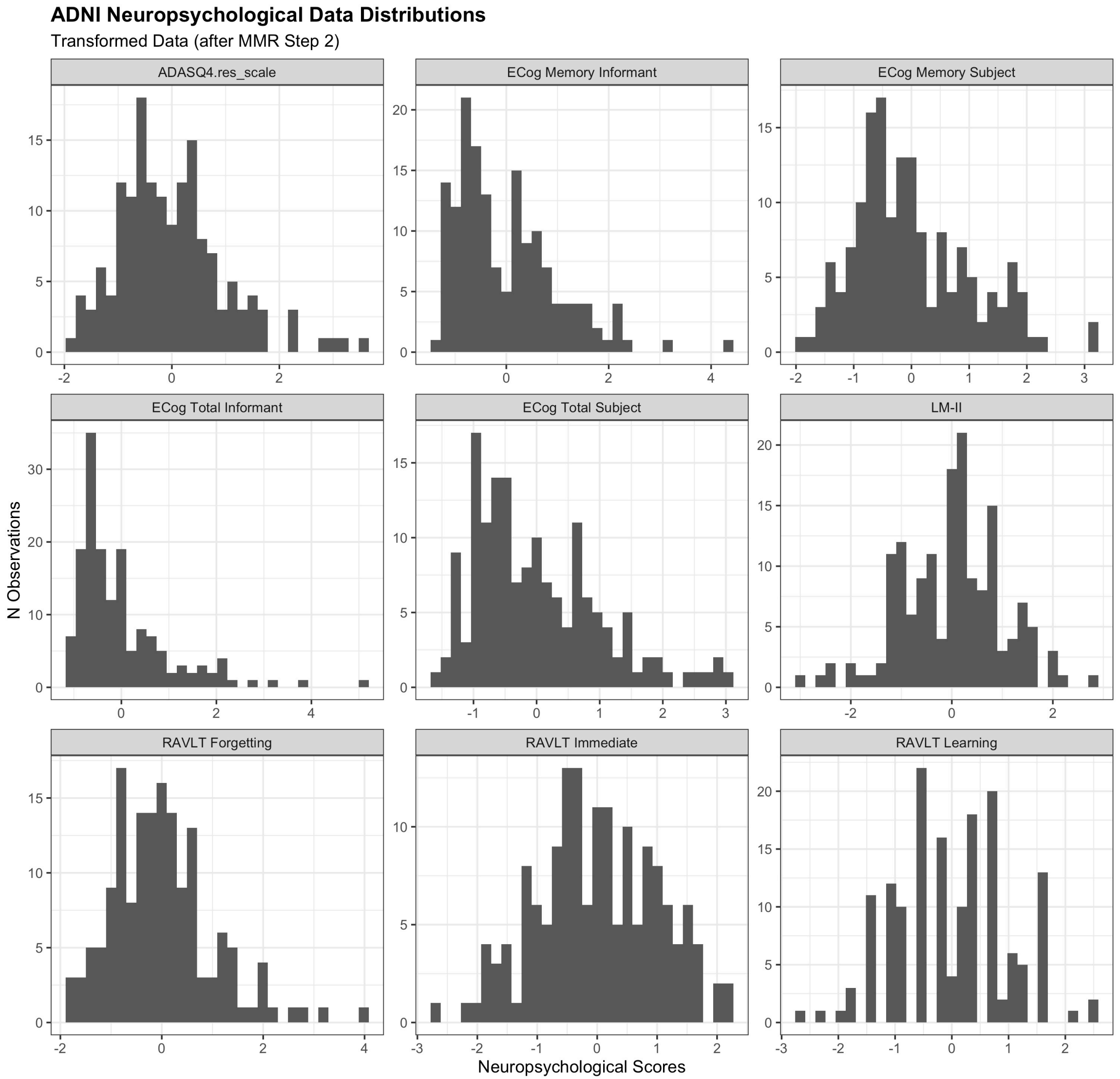

ECog = Everyday Cognition, RAVLT = Rey Auditory Verbal Learning Test,
ADAS = Alzheimer's Disease Assessment Scale, LM-II = Logical Memory Delayed

4. INSIGHT-PreAD, Neuropsychological Variables, Raw Distributions

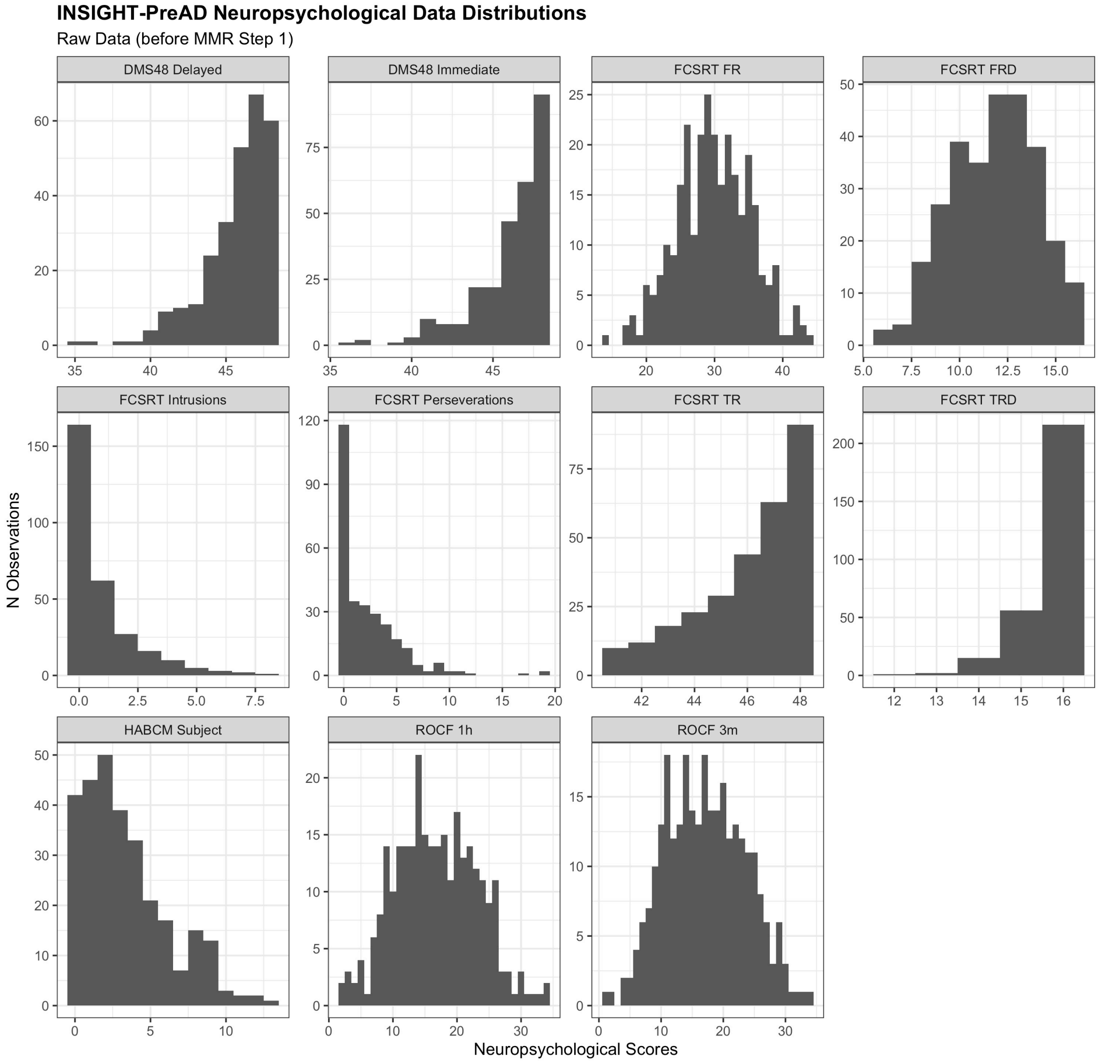

DMS48 = Delayed Matched to Sample 48 Items, FCSRT = Free and Cued Selective Remining Test,
ROCF = Rey-Osterrieth Complex Figure, FR(D) = Free Recall (Delayed), TR(D) = Total Recall (Delayed).

5. INSIGHT-PreAD, Neuropsychological Variables, Raw Distributions

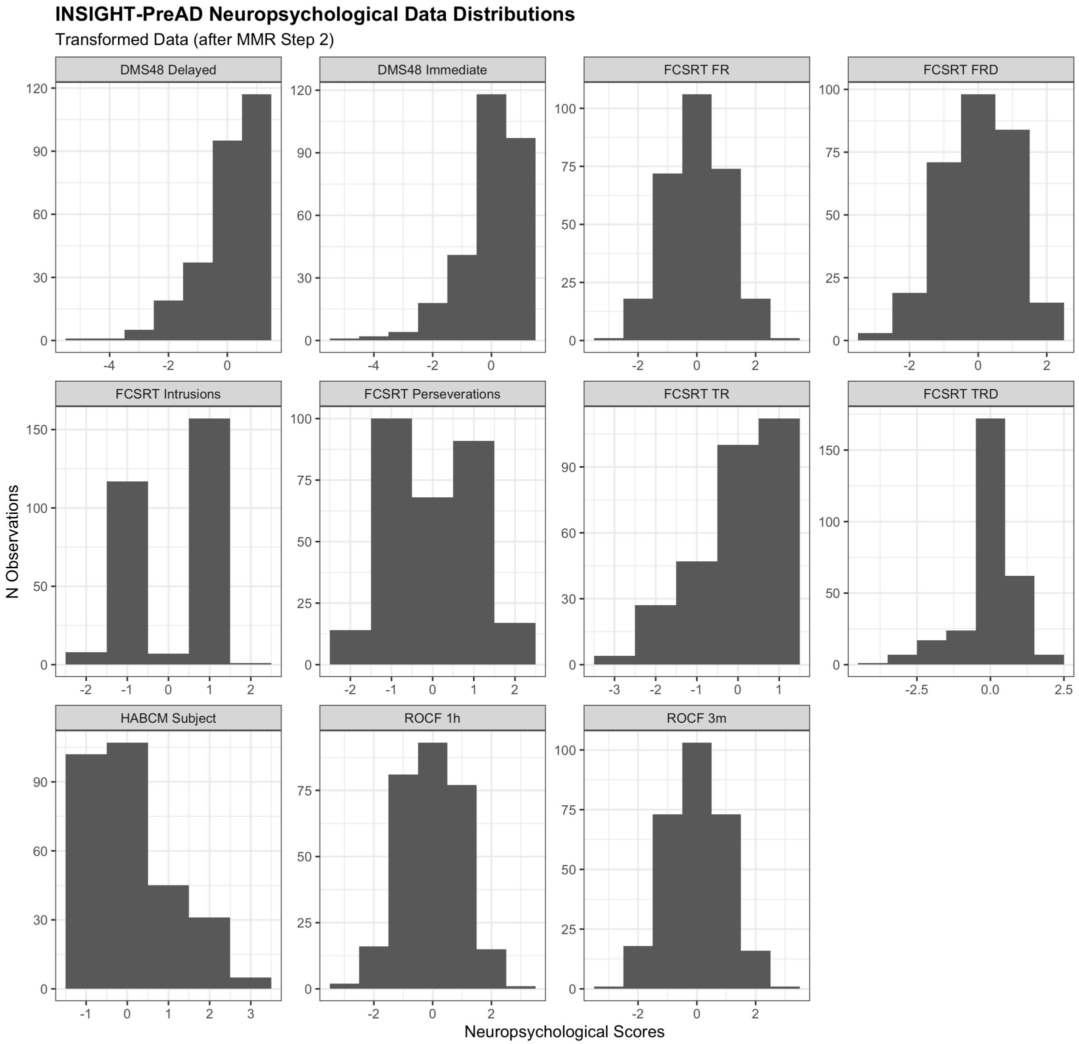

DMS48 = Delayed Matched to Sample 48 Items, FCSRT = Free and Cued Selective Remining Test, ROCF = Rey-Osterrieth Complex Figure, FR(D) = Free Recall (Delayed), TR(D) = Total Recall (Delayed).

6. Density of Merged Transformed Data

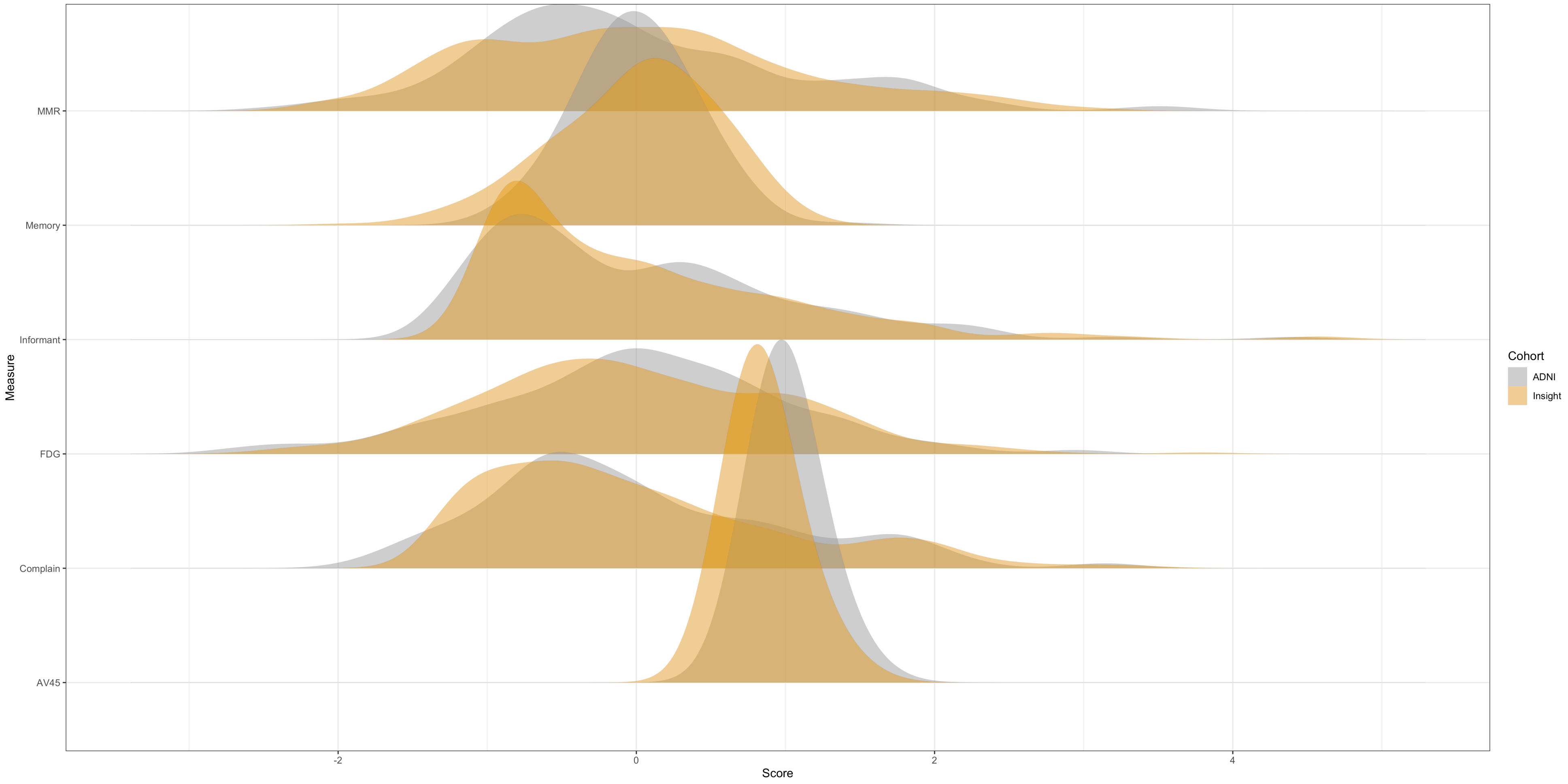

7. *By Cohort Biomarker vs Variables of Interest*

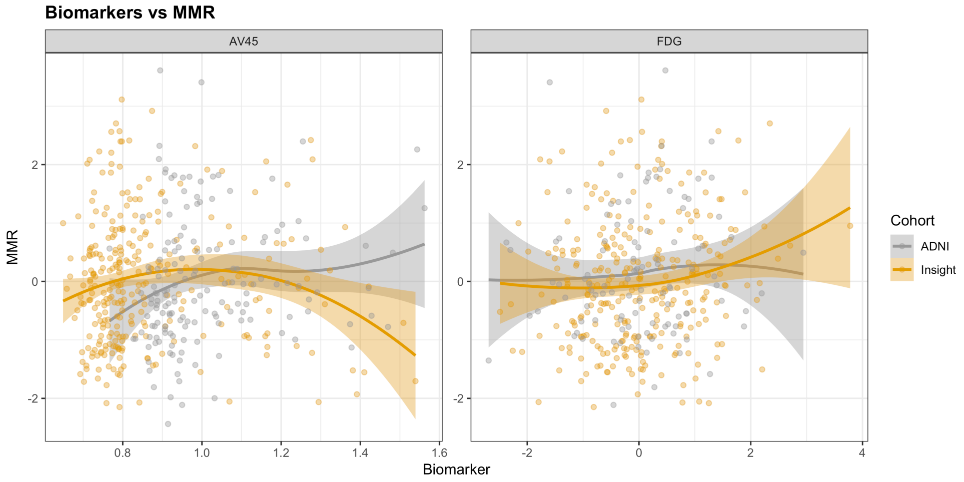

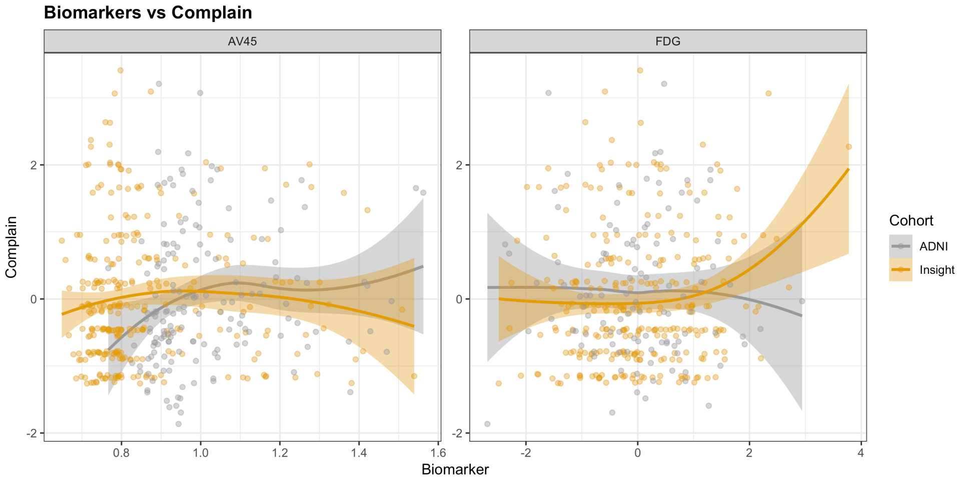

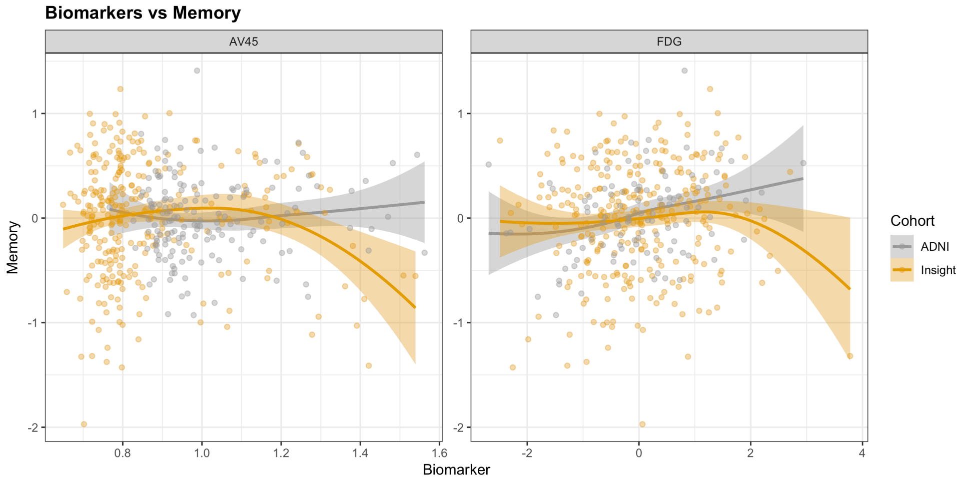

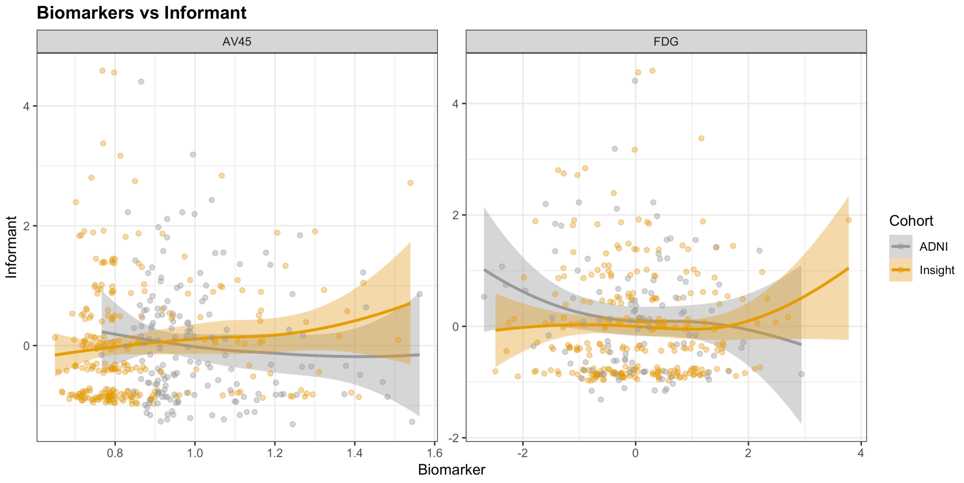

Supplement: Supplementary file 2 — Additional file 2. Data distribution of the samples. Population distribution regarding measures of interest (i.e. cognition, imaging and MMR). [file 13195_2020_626_MOESM2_ESM.pdf]
