# Supplementary material for: The meta-memory ratio: a new cohort-independent way to measure cognitive awareness in asymptomatic individuals at risk for Alzheimer’s disease
Source: Alzheimers Res Ther. 2020 May 14;12:57. doi: 10.1186/s13195-020-00626-1 (PMC7222501; doi:10.1186/s13195-020-00626-1)
Supplement: Supplementary file 3 — Additional file 3. Additional results for the presented model, and presentation of results with variations in the MMR construction. [file 13195_2020_626_MOESM3_ESM.pdf]

Additional Results

1. Complete results for the model

The following results correspond to the model presented in the article on MMR results. They also present the analyses performed on performance (objective and subjective, with the subject's and informant's complaint) standardized prior to the calculation of the MMR. As can be seen, the measures of complaints, whether from the patient himself or from the informant, do not seem to be affected by the various variables studied. On the other hand, memory performance seems to be significantly influenced by the cerebral amyloid concentration ($p = 0.013$, square effect).

	Complain			Informant			Memory			MMR		
Measures	Coefs ± SE	ESs	Pval	Coefs ± SE	ESs	Pval	Coefs ± SE	ESs	Pval	Coefs ± SE	ESs	Pval
Intercept	-2.72 ± 2.17	<0.001*		2 ± 2.23	<0.001*		-1.95 ± 1.06	<0.001*		-4.67 ± 2.3	<0.001*	
Age	0 ± 0.01	<0.001*	0.738	0 ± 0.01	<0.001*	0.882	-0.01 ± 0.01	0.010*	0.085	-0.01 ± 0.01	<0.001*	0.267
Gender (M)	0 ± 0.11	<0.001*	0.876	0 ± 0.11	<0.001*	0.992	0.02 ± 0.05	<0.001*	0.635	0.03 ± 0.12	<0.001*	0.714
Education (Lower)	0.13 ± 0.13	<0.001*	0.317	-0.07 ± 0.13	<0.001*	0.600	0 ± 0.06	<0.001*	0.951	0.13 ± 0.14	<0.001*	0.334
Cohort (ADNI)	0.75 ± 0.87	<0.001*	0.288	-1.45 ± 0.88	<0.001*	0.335	0.76 ± 0.43	<0.001*	0.783	1.51 ± 0.92	<0.001*	0.385
FDG	1.75 ± 1.3	<0.001*	0.215	0.35 ± 1.34	<0.001*	0.713	-0.79 ± 0.64	<0.001*	0.132	0.96 ± 1.38	<0.001*	0.063
AV45		0.001*	0.420		0.005*	0.232		-0.011*	0.013*		-0.007*	0.035*
Linear	5.42 ± 3.48	0.010*	0.454	-3.09 ± 3.58	<0.001*	0.399	4.9 ± 1.7	0.020*	0.529	10.31 ± 3.69	0.020*	0.681
Squared	-2.23 ± 1.51	0.010*	0.127	1.11 ± 1.55	<0.001*	0.477	-2.13 ± 0.74	0.020*	0.005*	-4.37 ± 1.6	0.020*	0.006*
AV45:FDG	1.5 ± 1.19	<0.001*	0.371	0.18 ± 1.22	<0.001*	0.680	-0.63 ± 0.58	<0.001*	0.181	0.87 ± 1.26	<0.001*	0.787
AV45:Cohort (ADNI)	-0.82 ± 0.86	<0.001*	0.597	1.33 ± 0.87	<0.001*	0.177	-0.7 ± 0.42	<0.001*	0.895	-1.52 ± 0.91	<0.001*	0.578
FDG:Cohort (ADNI)	0.05 ± 0.14	<0.001*	0.346	0.09 ± 0.14	<0.001*	0.593	-0.04 ± 0.07	<0.001*	0.782	0.01 ± 0.15	<0.001*	0.450

Table 1. Linear models results on behavioral variables using a composite memory score

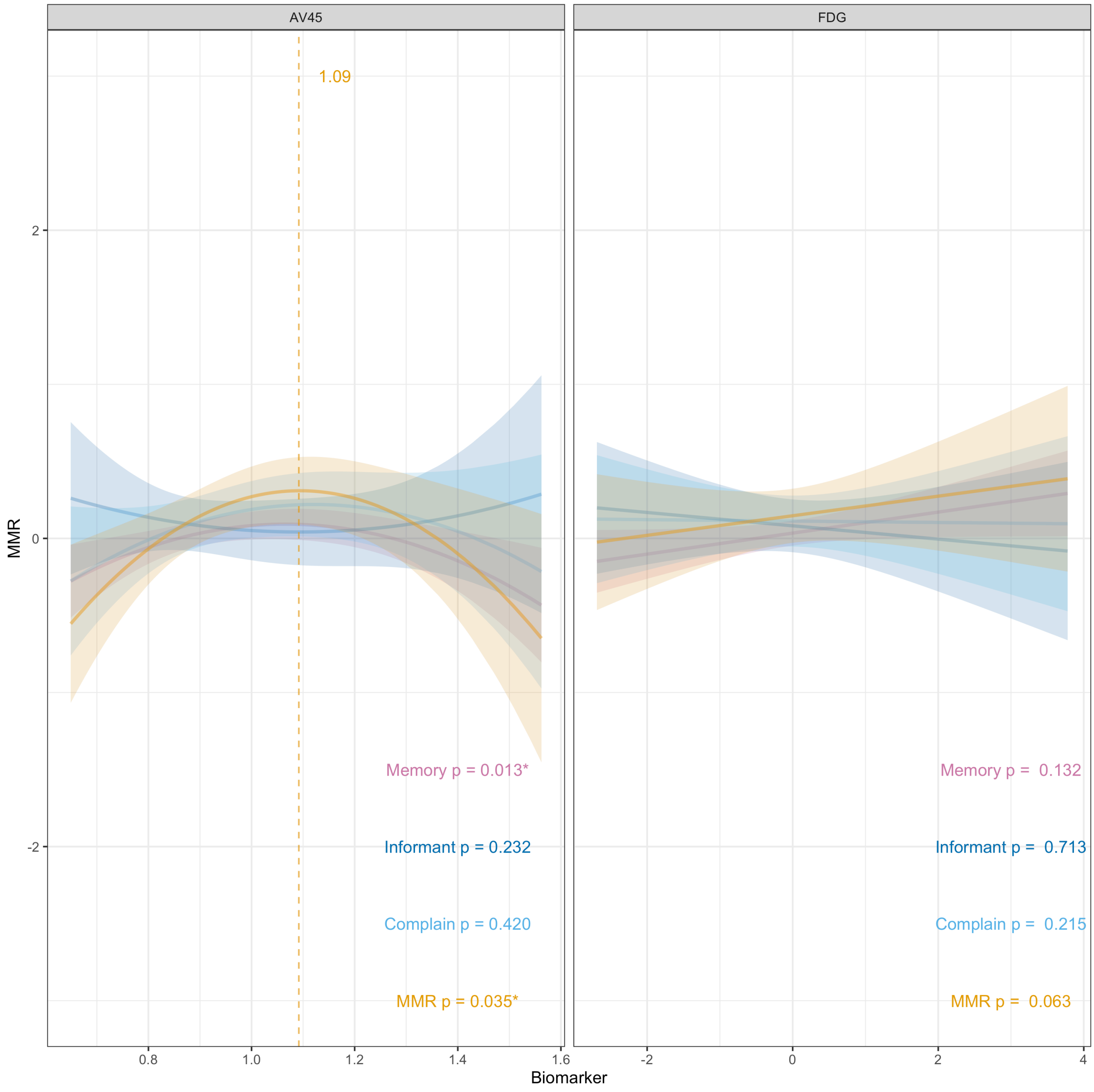

Figure 1. Effect of biomarkers on behavioral variables using a composite memory score

2. MMR computation with only one memory score

Here we present the MMR calculated from a single memory measurement. Indeed, it seems interesting to be able to perform this calculation not from a composite score, but from a single mnemonic measurement. This calculation could compensate for the numerical inequality of the memory tests proposed in the different cohorts available.

For this model, we used the Logical Memory Delayed Recall measure for ADNI, and the FCSRT Total Recall for INSIGHT-PreAD.

Nevertheless, with this model, the effect of the Aß does not persist (p = 0.077 instead of 0.029; FDG = 0.102 instead of 0.06).

It is possible that taking into account a single score does not account for the whole memory process. The design of a composite score, masking the various contingencies of each measure, could thus allow a more efficient and clean understanding of memory functioning.

Vars	ADNI (N = 158)	INSIGHT-PreAD (N = 290)	T/ChiSq	Pval
Age	71.97 ± 5.79	76.02 ± 3.5	-8.03	<0.001*
Gender (F)	95 (60.1%)	183 (63.1%)	0.27	0.604
Education (H)	157 (99.4%)	196 (67.6%)	59.94	<0.001*
AV45	1.01 ± 0.16 [0.77;1.56]	0.86 ± 0.17 [0.65;1.54]	9.42	<0.001*
FDG	0 ± 1 [-2.69;2.94]	0 ± 1 [-2.49;3.78]	0.00	1.000
Memory	0 ± 1 [-2.93;2.87]	0 ± 1 [-2.96;2.59]	0.00	1.000
Complain	0 ± 1 [-1.86;3.21]	0 ± 1 [-1.26;3.41]	0.00	1.000
Informant	0 ± 1 [-1.31;4.41]	0 ± 1 [-0.97;4.59]	0.00	1.000
MMR	0 ± 1.33 [-4.11;4.16]	0 ± 1.4 [-3.51;4.25]	0.00	1.000

Table 2. Differences between cohorts using a single memory score

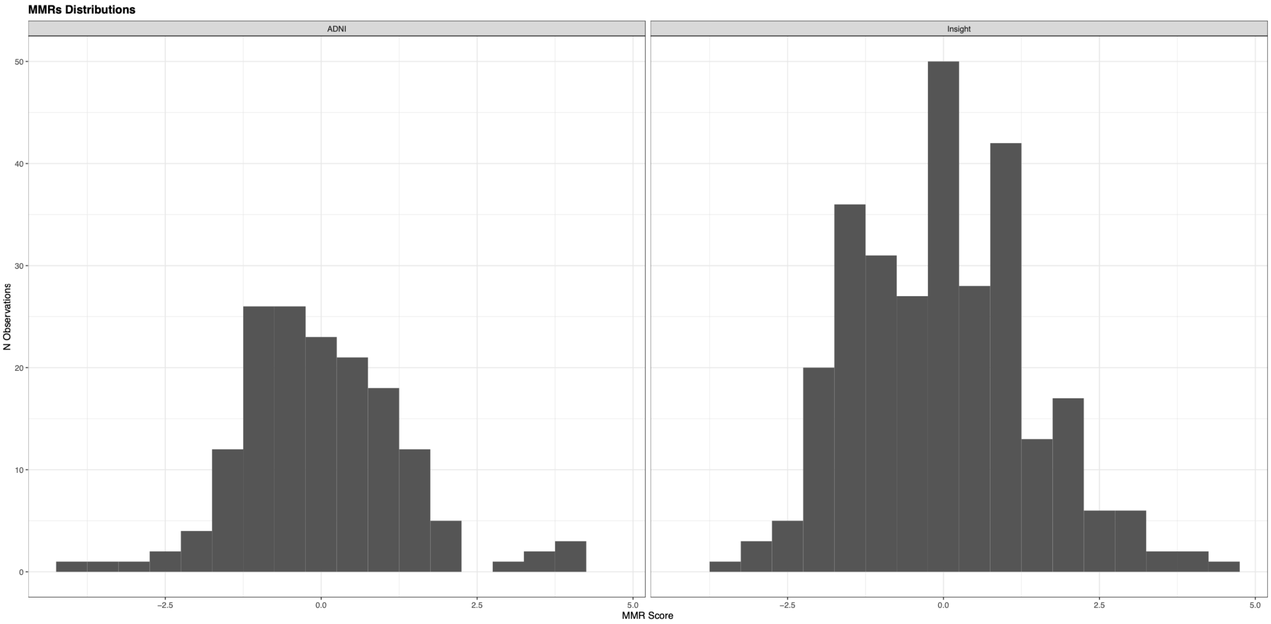

Figure 2. MMR score distribution using a single memory score.

	Complain			Informant			Memory			MMR		
Measures	Coefs ± SE	ESs	Pval	Coefs ± SE	ESs	Pval	Coefs ± SE	ESs	Pval	Coefs ± SE	ESs	Pval
Intercept	-2.72 ± 2.17	<0.001*		2 ± 2.23	<0.001*		1.66 ± 2.03	<0.001*		-1.06 ± 2.86	<0.001*	
Age	0 ± 0.01	<0.001*	0.738	0 ± 0.01	<0.001*	0.882	-0.03 ± 0.01	0.020*	0.006*	-0.04 ± 0.02	0.010*	0.028*
Gender (M)	0 ± 0.11	<0.001*	0.876	0 ± 0.11	<0.001*	0.992	-0.36 ± 0.1	<0.001*	0.001*	-0.36 ± 0.15	<0.001*	0.018*
Education (Lower)	0.13 ± 0.13	<0.001*	0.317	-0.07 ± 0.13	<0.001*	0.600	-0.2 ± 0.12	<0.001*	0.099	-0.07 ± 0.17	<0.001*	0.679
Cohort (ADNI)	0.75 ± 0.87	<0.001*	0.288	-1.45 ± 0.88	<0.001*	0.335	-0.14 ± 0.82	<0.001*	0.500	0.61 ± 1.15	<0.001*	0.743
FDG	1.75 ± 1.3	<0.001*	0.215	0.35 ± 1.34	<0.001*	0.713	-1.53 ± 1.22	<0.001*	0.332	0.22 ± 1.71	<0.001*	0.103
AV45		0.001*	0.420		0.005*	0.232		-0.059*	0.021*		-0.035*	0.114
Linear	5.42 ± 3.48	0.010*	0.454	-3.09 ± 3.58	<0.001*	0.399	2.53 ± 3.26	<0.001*	0.008*	7.95 ± 4.58	0.010*	0.186
Squared	-2.23 ± 1.51	0.010*	0.127	1.11 ± 1.55	<0.001*	0.477	-1.67 ± 1.41	<0.001*	0.258	-3.9 ± 1.99	0.010*	0.050*
AV45:FDG	1.5 ± 1.19	<0.001*	0.371	0.18 ± 1.22	<0.001*	0.680	-1.36 ± 1.12	<0.001*	0.367	0.14 ± 1.57	<0.001*	0.996
AV45:Cohort (ADNI)	-0.82 ± 0.86	<0.001*	0.597	1.33 ± 0.87	<0.001*	0.177	0.27 ± 0.81	<0.001*	0.203	-0.55 ± 1.13	<0.001*	0.615
FDG:Cohort (ADNI)	0.05 ± 0.14	<0.001*	0.346	0.09 ± 0.14	<0.001*	0.593	-0.02 ± 0.13	<0.001*	0.818	0.02 ± 0.18	<0.001*	0.581

Table 3. Linear models results on behavioral variables using a single memory score

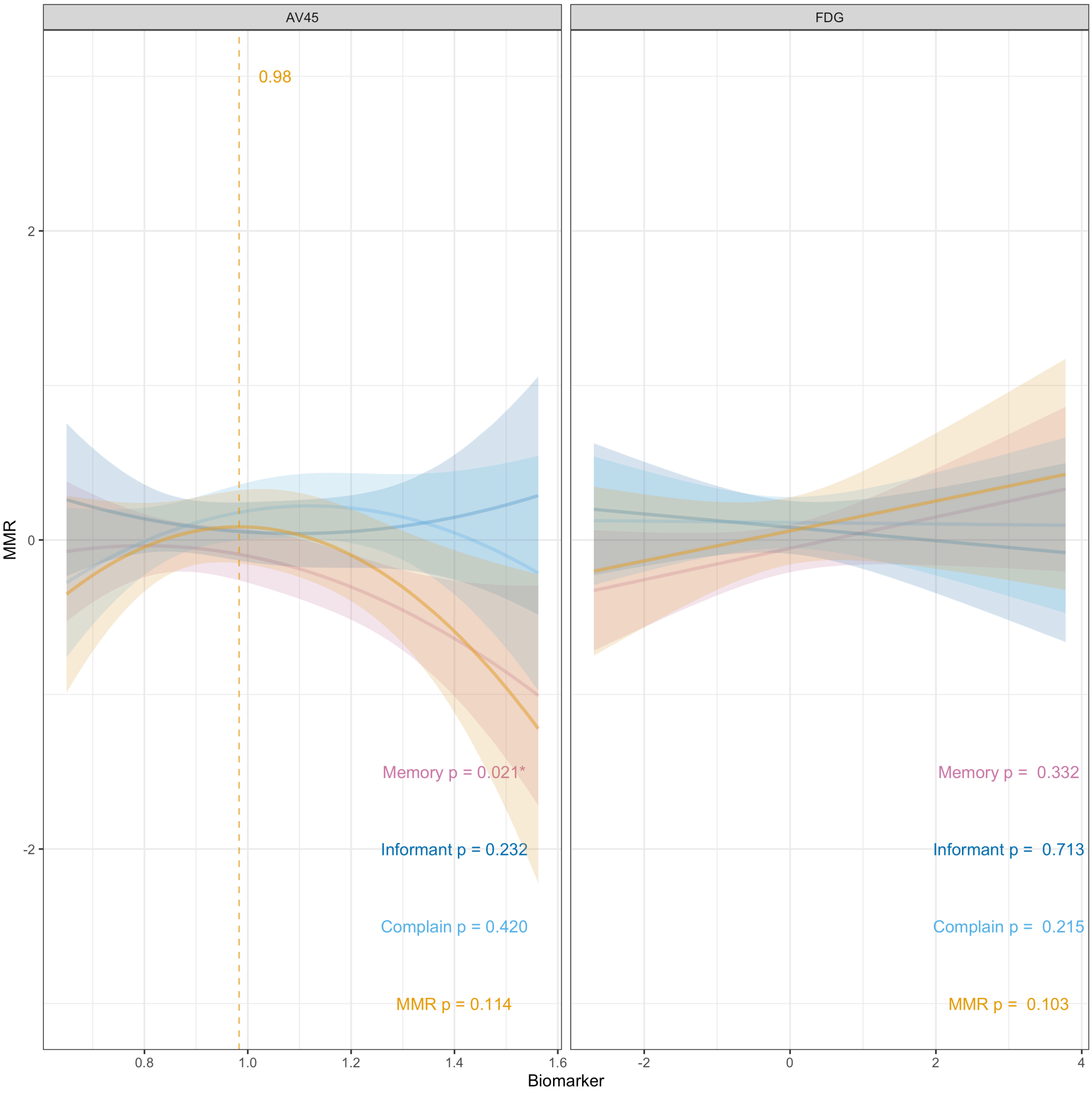

Figure 3. Effect of biomarkers on behavioral variables using a single memory score

3. MR computation with E-Cog Total Score

Here we present the MMR calculated with the same composite memory than in the article for objective measure. Regarding subjective measure, we used the HABC-M cognitive sub scale for INSIGHT-PreAD, and the E-Cog Total Score for ADNI instead of the Memory Subscale score in the article.

The results are very similar with those presented in the article. We observe a significant quadratic effect of Aß on ACD (p = 0.029 instead of 0.035), but only a trend regarding the metabolism (p = 0.060 instead of 0.063).

	ADNI (N = 158)	INSIGHT-PreAD (N = 290)	T/ChiSq	Pvalue
Age	71.97 ± 5.79	76.02 ± 3.5	-8.03	<0.001*
Gender (F)	95 (60.1%)	183 (63.1%)	0.27	0.604
Education (H)	157 (99.4%)	196 (67.6%)	59.94	<0.001*
AV45	1.01 ± 0.16 [0.77;1.56]	0.86 ± 0.17 [0.65;1.54]	9.42	<0.001*
FDG	0.00 ± 1.00 [-2.69;2.94]	0.00 ± 1.00 [-2.49;3.78]	0.00	1.000
Complain	0.00 ± 1.00 [-1.58;3.06]	0.00 ± 1.00 [-1.26;3.41]	0.00	1.000
Memory	0.00 ± 0.38 [-0.93;1.41]	0.00 ± 0.54 [-1.97;1.23]	0.04	0.970
MMR	0.00 ± 1.03 [-2.14;3.46]	0.00 ± 1.08 [-2.15;3.11]	0.02	0.988

Table 4. Differences between cohorts using a composite memory score and E-Cog Total score

Measures	Coefficients ± SE	ESs	Pvalue
Intercept	-4.82 ± 2.24	<0.001	
Age	-0.01 ± 0.01	<0.001	0.274
Gender (F)	0.05 ± 0.11	<0.001	0.609
Education (Higher)	0.13 ± 0.13	<0.001	0.330
Cohort (ADNI)	1.47 ± 0.9	<0.001	0.322
FDG	0.48 ± 1.34	<0.001	0.060
AV45		-0.007	0.029*
Linear	10.61 ± 3.6		
Squared	-4.55 ± 1.56		
AV45:FDG	0.36 ± 1.23	<0.001	0.953
AV45:Cohort (ADNI)	-1.49 ± 0.89	<0.001	0.699
FDG:Cohort (ADNI)	0.02 ± 0.14	<0.001	0.434

Table 4. Linear models results on behavioral variables using a composite memory score and E-Cog Total score

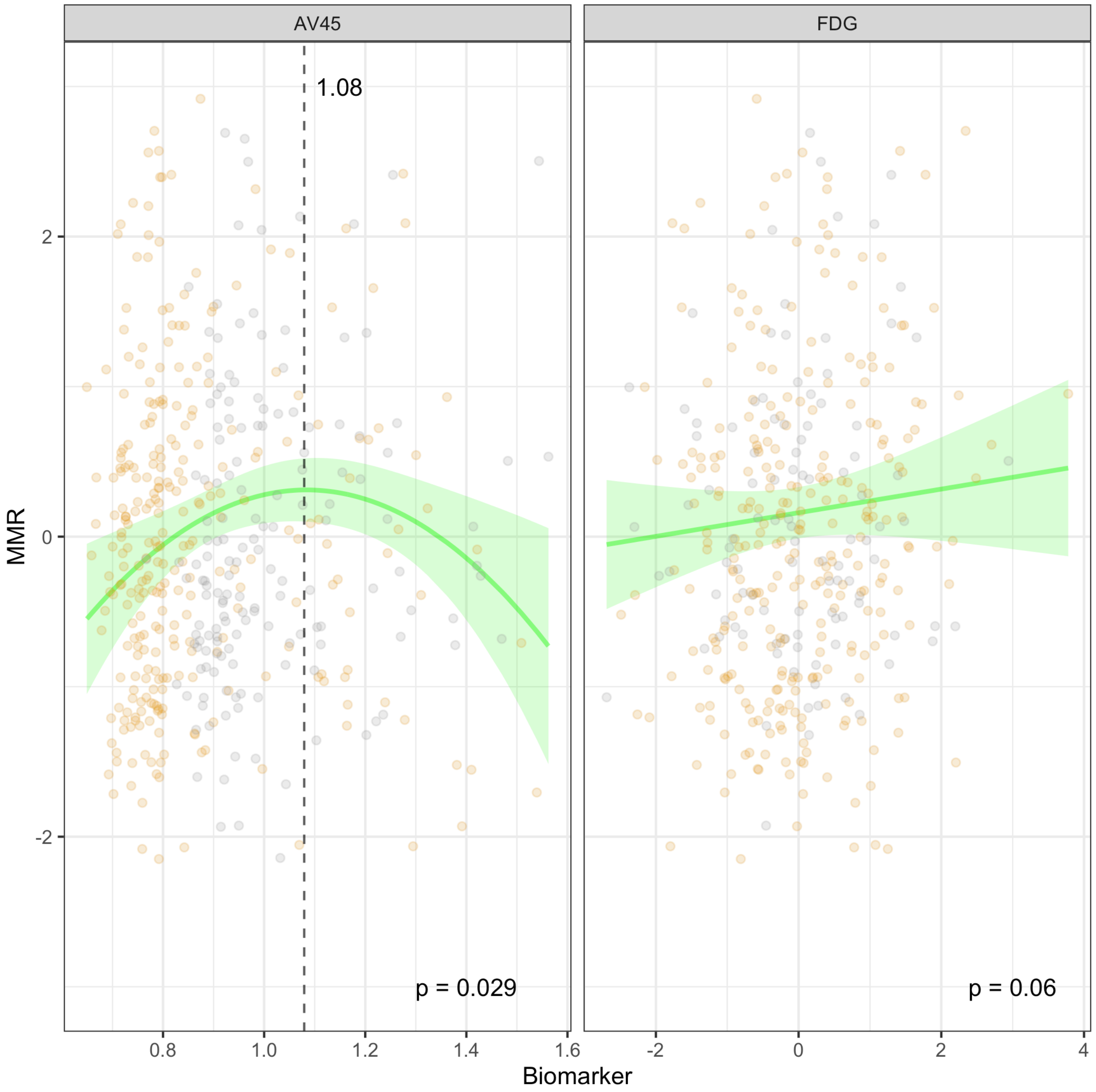

Figure 4. Effect of biomarkers on behavioral variables using a single memory score and E-Cog Total score
